# Editorial: Gluten, From Plant to Plate: Implications for People With Celiac Disease

**DOI:** 10.3389/fnut.2021.680418

**Published:** 2021-05-06

**Authors:** Michelle Lisa Colgrave, Katharina Anne Scherf, Melanie Downs, Alberto Caminero

**Affiliations:** ^1^Commonwealth Scientific and Industrial Research Organisation (CSIRO) Agriculture & Food, Brisbane, QLD, Australia; ^2^Department of Bioactive and Functional Food Chemistry, Institute of Applied Biosciences, Karlsruhe Institute of Technology (KIT), Karlsruhe, Germany; ^3^University of Nebraska-Lincoln, Food Science and Technology Department, Lincoln, NE, United States; ^4^Faculty of Health Sciences, McMaster University, Hamilton, ON, Canada

**Keywords:** gluten, celiac disease, cereal breeding, wheat, food processing

Gluten is the collective name for a class of proteins found in wheat, rye, and barley. Eating gluten triggers an autoimmune reaction in the ~70 million people globally affected by celiac disease (CD), which causes the gut to react to gluten with intestinal inflammation and epithelial cell damage. In addition, wheat proteins may trigger respiratory, skin or food allergies and non-celiac gluten/wheat sensitivity (NCGS). Recently, more and more evidence has been emerging to support an increasing prevalence of gluten-related disorders in the population. This increase in prevalence has been too quick to be explained by genetic drift, pointing toward a change in environmental exposures as risk modifiers.

Gluten-free (GF) foods are now commonplace, offering consumers greater choice and availability. While many of these foods are made from non-gluten-containing grains, contamination of these inherently gluten-free products can occur during harvest, transport, or processing. Moreover, these foods are expensive and may be nutritionally inferior to gluten-containing products. The differences in nutritional properties of GF foods has led to research on ways to remove or reduce gluten from wheat and barley to provide new fiber, mineral, and vitamin options for those who must avoid gluten. This has led to research in classical plant breeding and the use of gene technology. An alternative approach to producing celiac-safe foods is via processing, wherein processes, such as separation, filtration, and/or application of enzymes, aim to remove gluten from gluten-containing ingredients. With many of these processed products entering the market, questions remain over the safety of these products and controversy over a suitable test to determine the gluten content remains. The question why an increasing number of people are affected by gluten-related disorders also needs to be answered. While improved diagnostics and awareness may partly explain the rise, further factors such as the use of vital wheat gluten in many food products, changes in wheat processing and in wheat protein composition may be responsible. In addition, it has been proposed that other environmental factors such as introduction of gluten to infant diets, breastfeeding patterns, alterations in the gut microbiota and infections could also dictate the development of gluten-related disorders.

This e-book is a compilation of 15 research and/or review papers written by 70 authors. This Special Research Topic comprises contributions from leading experts in the fields of plant breeding, food processing, clinical immunology, and gluten analysis to share their latest findings and help improve the quality and safety of foods for CD patients and other gluten-related disorders.

As critical overviews of the field, Shewry reviews wheat gluten, focusing on functional properties, and its role in triggering coeliac disease and gluten-related disorders. Wieser et al. provide a balanced review on the benefits of wheat consumption contrasted with the adverse effects for individuals suffering from wheat-related disorders.

Starting with the plant, Tanner et al. examined the accumulation of hordein storage proteins in developing barley grains using a combination of enzyme-linked immunosorbent assay (ELISA), western blot and liquid chromatography tandem mass spectrometry (LC-MS/MS) demonstrating maximum protein accumulation late in grain development. Shifting to wheat, Altenbach, Chang, Yu et al. described the genetic transformation of bread wheat to reduce the omega-1,2 gliadins, the wheat gluten proteins that present immunodominant epitopes relevant in celiac disease. Subsequently, the same group silence a subset of the alpha-gliadins and demonstrate reduced reactivities of antibodies (IgG and IgA) from a celiac disease patient cohort. Marín-Sanz et al. explore the impact of temperature and nitrogen availability of grain filling in bread wheat focusing specifically on the gliadin and glutenin protein fractions.

Shifting to gluten analysis, Panda and Garber review the use of antibody-based methods for accuracy in the quantitation of gluten in fermented or hydrolyzed foods and the inherent challenges due to the lack of appropriate reference materials and variable proteolysis. Next Alves et al. review the primary proteomic approaches used in the identification and quantitation of gluten peptides related to CD-activity and gluten-related allergies. In a complementary study, Daly et al. describe an update to the GluPro database, that provides a solid foundation for proteomic analysis of gluten proteins from gluten containing cereals. This database will enable identification of peptide markers for use in new gluten quantitation methods based on coeliac toxic motifs present in all relevant cereal species. To this end, Lexhaller et al. characterize and quantify cereal-specific gluten protein types by LC-MS/MS, allowing known wheat allergens and celiac disease-active peptides to be identified and laying the foundation for development of reference materials. Subsequently, five wheat cultivars were assessed by Schall et al. for their use as reference materials wherein their protein content, protein composition and responses to different ELISA methods were evaluated.

Osorio et al. examine the ability to detoxify gluten proteins using “glutenases” and employing site-directed mutagenesis aimed at the glutamine specific endoprotease from barley (EP-B2), and a prolyl endopeptidase from *Flavobacterium meningosepticum* (Fm-PEP).

From a clinical perspective, Pinto-Sanchez and Bai review the current strategies for follow up of patients with celiac disease, describing new tools for monitoring adherence to the gluten-free diet which could alter patient treatment. The isolation and purification of oat avenin for clinical trials aiming to establish the safety of oats in the diets of those with CD is reported by Tanner et al. Lastly, the Prolamin Working Group provide recommendations regarding clinical, analytical and legal aspects of CD, identifying those areas that require future multidisciplinary collaborative efforts.

## Author Contributions

MC prepared the initial draft concept of the Special Topic. MC, KS, MD, and AC refined the topic, compiled a list of potential contributing authors, and were associate editors for all submitted manuscripts. All authors contributed to the article and approved the submitted version.

## Conflict of Interest

The authors declare that the research was conducted in the absence of any commercial or financial relationships that could be construed as a potential conflict of interest.

